# Lutein acts via multiple antioxidant pathways in the photo-stressed retina

**DOI:** 10.1038/srep30226

**Published:** 2016-07-22

**Authors:** Mamoru Kamoshita, Eriko Toda, Hideto Osada, Toshio Narimatsu, Saori Kobayashi, Kazuo Tsubota, Yoko Ozawa

**Affiliations:** 1 Laboratory of Retinal Cell Biology, Keio University School of Medicine, 35 Shinanomachi, Shinjuku-ku, Tokyo 160-8582, Japan; 2 Department of Ophthalmology, Keio University School of Medicine, 35 Shinanomachi, Shinjuku-ku, Tokyo 160-8582, Japan; 3 Wakasa Seikatsu Co., Ltd., 134 Chudoujiminami-cho, Shimogyo-ku, Kyoto 600-8813, Japan

## Abstract

Lutein slows the progression of age-related macular degeneration (AMD), a leading cause of blindness in ageing societies. However, the underlying mechanisms remain elusive. Here, we evaluated lutein’s effects on light-induced AMD-related pathological events. Balb/c mice exposed to light (2000 lux, 3 h) showed tight junction disruption in the retinal pigment epithelium (RPE) at 12 h, as detected by zona occludens-1 immunostaining. Substantial disruption remained 48 h after light exposure in the vehicle-treated group; however, this was ameliorated in the mice treated with intraperitoneal lutein at 12 h, suggesting that lutein promoted tight junction repair. In the photo-stressed RPE and the neighbouring choroid tissue, lutein suppressed reactive oxygen species and increased superoxide dismutase (SOD) activity at 24 h, and produced sustained increases in *sod1* and *sod2* mRNA levels at 48 h. SOD activity was induced by lutein in an RPE cell line, ARPE19. We also found that lutein suppressed upregulation of macrophage-related markers, *f4/80* and *mcp-1*, in the RPE-choroid tissue at 18 h. In ARPE19, lutein reduced *mcp-1* mRNA levels. These findings indicated that lutein promoted tight junction repair and suppressed inflammation in photo-stressed mice, reducing local oxidative stress by direct scavenging and most likely by induction of endogenous antioxidant enzymes.

Age-related macular degeneration (AMD) is currently the leading cause of blindness in ageing societies. There are two subtypes of this condition: wet AMD, which inflicts permanent vision damage in spite of therapeutic interventions, and dry AMD, which has no specific treatment and causes gradual visual loss. This lack of satisfactory therapy has increased the interest in preventive approaches to AMD. Large clinical studies of preventive therapies, the Age-related Eye Disease Study (AREDS)^1^ and AREDS2^2^, have been performed. AREDS2 identified lutein^3^, a xanthophyll carotenoid and oral antioxidant nutrient supplement that is delivered via the circulation^4^, to enhance the beneficial effects of the multi-vitamins and zinc that were proven to be effective in the original AREDS. Moreover, the protective effect of lutein intake on the photo-stressed retina was demonstrated by fundus findings in rhesus monkeys^5^. However, the mechanism underlying this effect remains to be elucidated.

AMD is fundamentally related to stress-induced changes in the retinal pigment epithelium (RPE)^6^, which constitutes the blood-retinal barrier and regulates this microenvironment. Risk factors for AMD such as smoking, metabolic syndrome that raises body mass index, and light exposure^7^,^8^ may increase RPE stress. Excessive light exposure causes oxidative stress^3^,^9^,^10^,^11^,^12^,^13^, at least in part by inducing increased activation of the visual cycle in the retina^14^,^15^. Therefore, lutein’s effects on pathological events in the photo-stressed retina are of considerable interest to researchers considering AMD risk.

Exposure of the RPE to excessive light has been previously reported to disrupt tight junctions^12^. These provide the blood-retinal barrier that separates the neural retina from the choroidal vascular tissue; tight junction disruption is one of the critical features of AMD pathogenesis^16^ because it increases macrophage invasion, inflammatory cytokine levels, and choroidal neovascularization^15^,^16^. Light exposure also induces production of monocyte chemotactic protein-1 (MCP-1)^9^,^12^,^17^, an inflammatory cytokine that is critical for AMD owing to its macrophage recruiting effects^18^,^19^ in the RPE and/or the choroid.

In this study, we evaluated the effects of lutein on these cellular events in the photo-stressed retina to determine whether lutein could repair the blood-retinal barrier and suppress inflammatory events. Moreover, we explored the mechanisms underlying these effects by investigating the levels of reactive oxygen species (ROS). ROS levels can be reduced by free radical scavengers and by endogenous antioxidant enzymes. Lutein has many double bonds in its chemical structure and can therefore act as a direct scavenger^3^; we also analysed whether this compound affected endogenous antioxidant enzymes. The current results may improve understanding of the value of lutein usage in suppressing oxidative stress in the RPE-choroid, which determines the risk for AMD.

## Results

### Lutein ameliorated photo-induced disruption of RPE tight junctions

**Figure 1:**
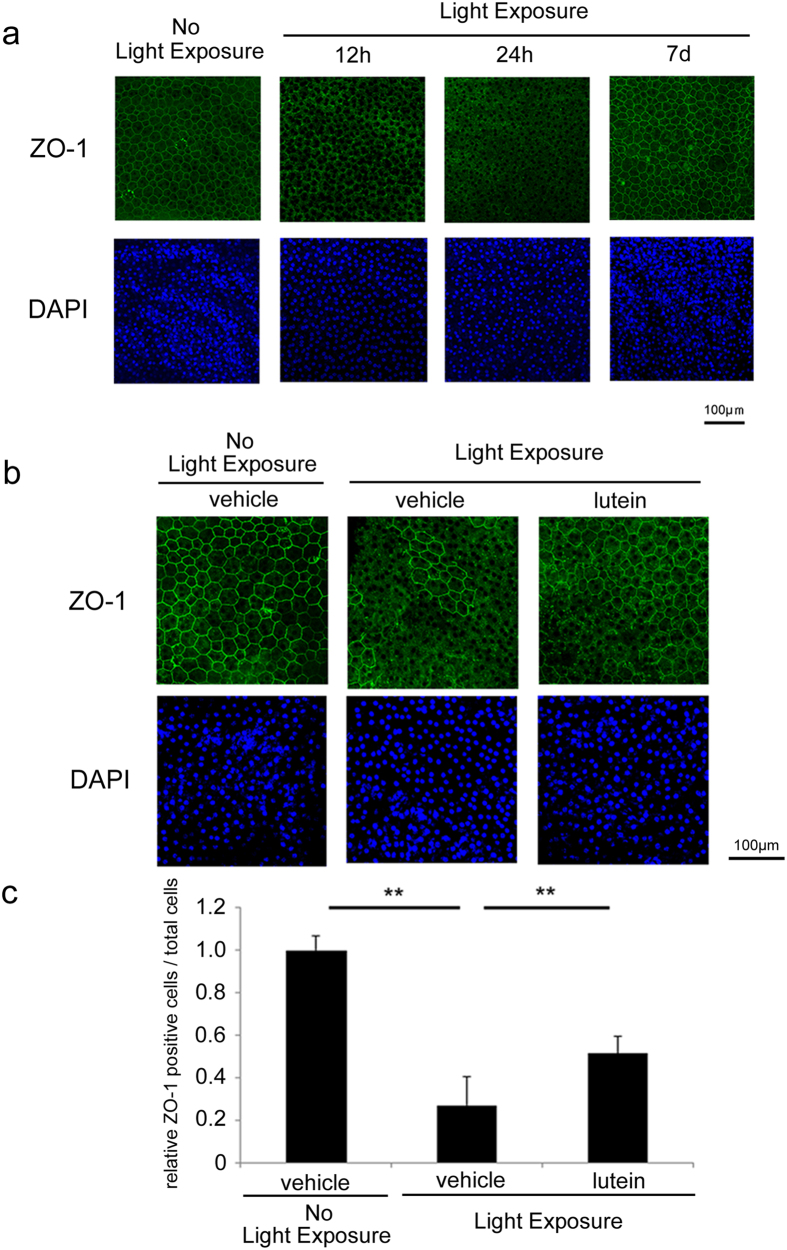
Repair of photo-induced tight junction disruption was promoted by lutein. Whole mount RPE immunostaining for ZO-1 and counterstaining using Hoechst. (**a**) Light exposure disrupted the ZO-1 (green) staining pattern in the RPE at both 12 and 24 h; this disruption was reduced at 7 days. Hoechst (blue) showed no obvious change during this time-course. (**b**) At 48 h, the disruption of the ZO-1 pattern was attenuated by lutein treatment at 12 h, as compared with vehicle treatment. (**c**) The number of RPE cells with an intact ZO-1 pattern at all edges of the RPE cells per total RPE cells were shown in a graph. RPE, retinal pigment epithelium; n = 6; **p < 0.01. Scale bar, 100 μm.

Photo-induced tight junction disruption was detected by zona occludens-1 (ZO-1) immunostaining 12 h after light exposure and was still evident at 24 h (Fig. 1a). ZO-1 is typically observed on the intracellular face of the entire cell membrane, but it was dissociated from the membrane after light exposure. The tight junctions were gradually and spontaneously repaired at 7 days after the single light exposure employed in this study (Fig. 1a). To evaluate the effects of lutein, mice were intraperitoneally injected with lutein 12 h after light exposure, and ZO-1 immunostaining was evaluated in the flat mount samples at 48 h (Fig. 1b,c). Our evaluation of the proportion of the RPE cells that showed intact expression of ZO-1 at the entire cell membrane found that lutein treatment succeeded in promoting tight junction repair, as compared with vehicle-treated mice. During the study time-period, there was no obvious nuclear condensation in the RPE cells of animals exposed to light and treated with either vehicle or lutein, suggesting a lack of RPE cell death.

### Lutein suppressed ROS levels in the photo-stressed RPE-choroid

**Figure 2:**
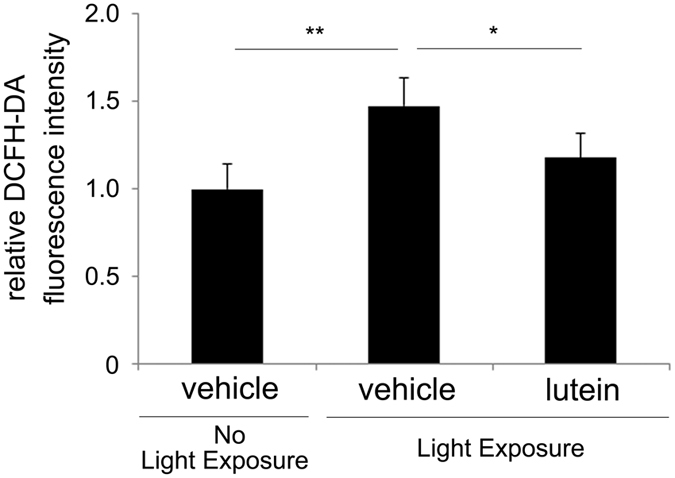
ROS levels were suppressed by lutein in the photo-stressed RPE-choroid. ROS levels, as indicated by DCFH-DA fluorescent intensity, were attenuated by lutein treatment 24 h after light exposure in the photo-stressed RPE-choroid. RPE, retinal pigment epithelium; n = 6; **p < 0.01.

Next, we measured ROS levels in the complex samples of the RPE and the neighbouring choroid tissue, because they could not be separated for technical reasons. Dichlorodihydrofluorescein diacetate (DCFH-DA), which becomes fluorescent when reacted with hydroxyl and peroxyl compounds and other ROS, was added to the RPE-choroid complex sample prior to measuring the fluorescence intensity as previous reported^9^,^12^. As predicted by its chemical structure, lutein-treated mice showed lower ROS levels in the RPE-choroid complex 24 h after light exposure, than vehicle-treated mice (Fig. 2).

### Lutein induced endogenous antioxidant enzymes in the photo-stressed RPE-choroid

**Figure 3:**
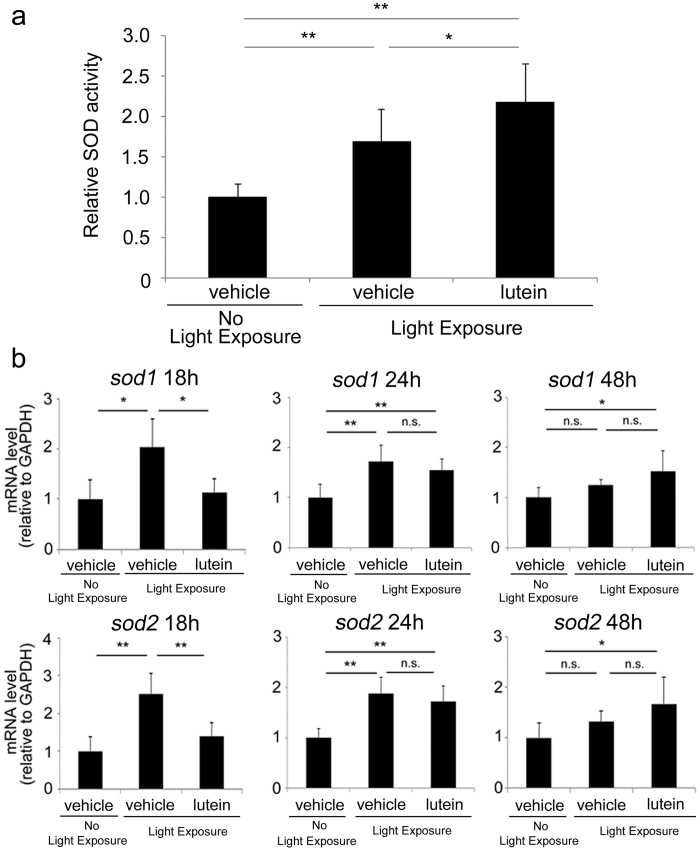
Lutein induced SOD activity and mRNA expression in the photo-stressed RPE-choroid. (**a**) Light exposure induced SOD activity in the RPE-choroid 24 h after light exposure; this induction was greater in lutein-treated mice. (**b**) Levels of *sod1* and *sod2* mRNAs, as determined by real-time RT-PCR. RPE, retinal pigment epithelium; n = 6; *p < 0.05, **p < 0.01.

We measured the activities of the endogenous antioxidant enzymes, superoxide dismutases 1 and 2 (SOD1 and SOD2), 24 h after light exposure. In general, these enzymes are induced in response to ROS accumulation^20^, and their activities increased in the photo-stressed RPE-choroid samples. SOD activity was elevated in mice treated with lutein, as compared with those treated with vehicle (Fig. 3a).

In addition, lutein treatment was associated with upregulation of *sod1* and *sod2* mRNA levels in the photo-stressed RPE-choroid (Fig. 3b). At 18 h after light exposure, both *sod1* and *sod2* mRNAs were induced in vehicle-treated group, but not in the lutein treatment group (Fig. 3b). However, both mRNAs were induced in the presence or absence of lutein treatment by 24 h after light exposure compared with non-light exposed vehicle treatment group (Fig. 3b). Interestingly, these levels remained elevated in the lutein treatment group 48 h after light exposure and there still existed a significant difference compared with non-light exposed vehicle treatment group, while in the light-exposed vehicle-treatment group, the mRNA levels had almost been returned to the basal levels and similar levels to the non-light exposed vehicle treatment group (Fig. 3b). These findings suggested that lutein treatment sustained the expression of *sod1* and *sod2* for a longer period in the photo-stressed RPE-choroid.

### Lutein induced antioxidant enzymes in ARPE19 cells

**Figure 4:**
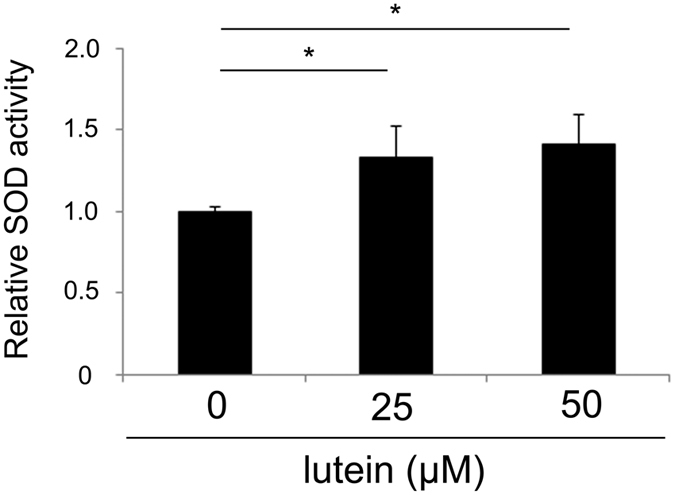
Lutein induced SOD activity in the ARPE19 cell line. SOD activity is shown in the ARPE19 cell line 3 h after exposure to the indicated concentrations of lutein; n = 4; *p < 0.05, **p < 0.01.

The *in vivo* samples analysed above included both RPE and choroid. To investigate whether lutein affected RPE cells, we measured its effects on SOD activity in an RPE cell line, ARPE19. Lutein induced a concentration-dependent increase in SOD activity, which was observed 3 h after lutein treatment (Fig. 4).

### Lutein suppressed macrophage recruitment and mcp-1 expression in the photo-stressed RPE-choroid

**Figure 5:**
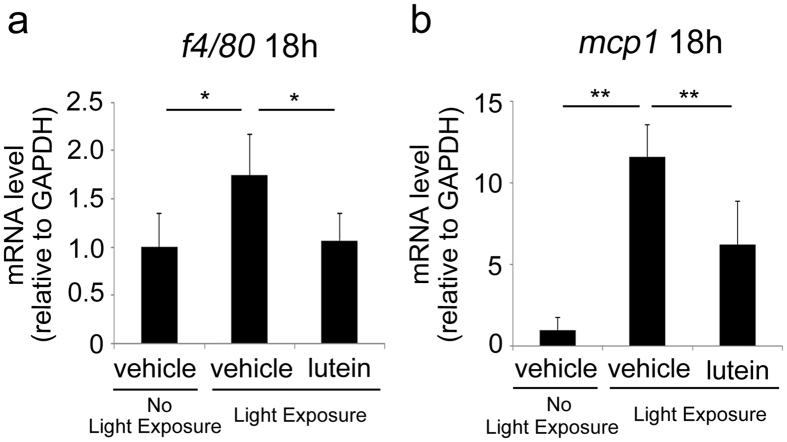
Lutein suppressed macrophage recruitment in the photo-stressed RPE-choroid. Photo-stress induced *f4/80* (**a**) and *mcp-1* (**b**) mRNAs, as determined by real time RT-PCR, at 18 h; lutein treatment suppressed this induction. RPE, retinal pigment epithelium; n = 6; *p < 0.05, **p < 0.01.

Macrophage recruitment is critical in AMD pathogenesis^15^,^18^ and is induced in the photo-stressed RPE-choroid, as reported previously^9^,^12^. We measured *f4/80* mRNA levels in photo-stressed RPE-choroid samples at 18 h and found that lutein treatment suppressed the light-induced increase in the *f4/80* mRNA level (Fig. 5a). This suggested that lutein treatment suppressed photo-induced macrophage recruitment. In addition, the level of mRNA encoding *mcp-1*, a macrophage-recruiting factor, was also suppressed in the RPE-choroid of lutein-treated mice 18 h after light exposure (Fig. 5b).

### Lutein reduced mcp-1 expression in ARPE19 cells

**Figure 6:**
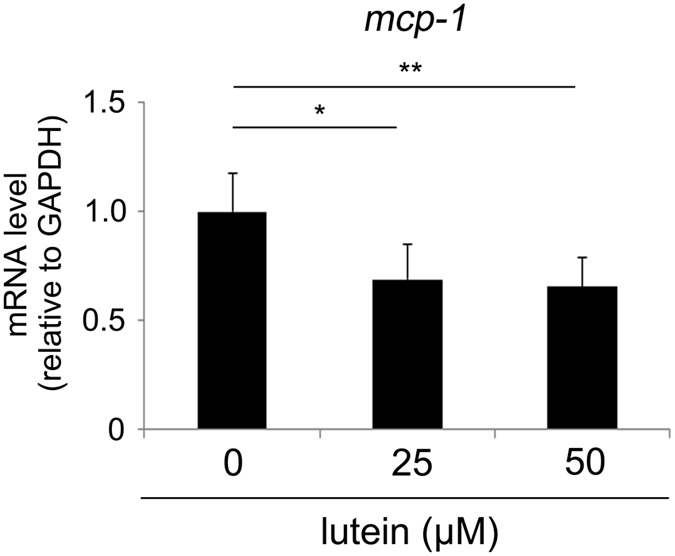
Lutein suppressed *mcp-1* mRNA in the ARPE19 cell line. Lutein reduced *mcp-1* mRNA levels in the ARPE19 cell line, as determined by real-time RT-PCR, in a concentration-dependent manner 3 h after addition of the indicated concentrations of lutein to the culture medium; n = 6; *p < 0.05, **p < 0.01.

We also analysed whether lutein suppressed *mcp-1* mRNA expression in the ARPE19 RPE cell line. Interestingly, lutein reduced *mcp-1* mRNA levels in this cell line at 3 h (Fig. 6), suggesting that lutein may have attenuated light-induced *mcp-1* expression in the RPE.

## Discussion

The present study demonstrated that lutein treatment promoted repair of photo-induced tight junction disruption (Fig. 1). Lutein suppressed ROS levels (Fig. 2) and increased activity of the endogenous antioxidant SODs, as well as produced a more sustained increase in their mRNA levels in the RPE-choroid of light-exposed mice (Fig. 3). SOD activity was also induced in ARPE19 cells exposed to lutein (Fig. 4). Light-induced *mcp-1* and the resulting *f4/80*-positive macrophage recruitment were also suppressed by lutein in the photo-stressed RPE-choroid (Fig. 5), and the expression of *mcp-1* mRNA decreased in ARPE19 cells exposed to lutein (Fig. 6).

Light exposure disrupted tight junctions, which are indispensable for the barrier function of the RPE^16^,^21^,^22^,^23^. Tight junctions can be disrupted by ROS^23^, as well as by activation of Rho-Rho-associated protein kinase (Rho-ROCK)^24^,^25^ and protein kinase C^26^; multiple pathways can be involved in this process. Lutein suppressed this disruption and reduced ROS levels, suggesting that its effects were, at least in part, mediated by antioxidant activity. We have previously reported that ROS initiates RPE barrier disruption, activating Rho-ROCK signalling^12^. In the present study, the time-point of lutein administration was 12 h after light exposure, when ROS accumulated to activate downstream signalling and most of the tight junctions had already been disrupted. Thus, multiple downstream signalling molecules would already have been activated^12^; however, ROS suppression by lutein succeeded in repairing the barrier, suggesting that this process was still regulated by ROS. The current findings therefore indicated that in addition to initiating photo-induced barrier disruption^12^, ROS were involved in sustaining this disruption. Lutein was effective, even when administered after barrier disruption, raising the possibility that it could show efficacy in patients with early AMD and in other older people with an elevated risk for AMD, who may already have sustained some photo-induced oxidative stress and tissue damage.

Previous reports showed that tight junctions were disrupted in other AMD models. Interleukin-18, which increased in the serum of dry AMD-induced tight junction disruption in the RPE of mice^27^. Aryl hydrocarbon receptor-deficient mice, which show AMD characteristics such as accumulation of RPE lipofuscin and Bruch’s membrane thickening, also showed tight junction disruption^28^. It would be interesting to evaluate whether tight junction disruption, a finding closely related to AMD, can also be regulated by ROS and ameliorated by lutein in these models.

Lutein is likely to reduce ROS levels by scavenging and by inducing the activity of SOD antioxidant enzymes, which are generally induced in response to accumulated ROS. Lutein treatment was associated with increased SOD activity, even though ROS levels were reduced, suggesting that lutein acted as a ROS-independent SOD activator. This was consistent with our observation that ARPE19 SOD activity was induced by lutein in the absence of other stimuli, indicating that lutein directly induced SOD activity.

Moreover, mRNA transcripts of *sod1* and *sod2* were upregulated *in vivo* under conditions where ROS levels were reduced, even though the expression of antioxidant enzymes is generally upregulated by ROS via the stabilization of a transcription factor, Nrf2^29^. However, this unique phenomenon was consistent with our previous findings in a neuronal cell line, PC12D cells^30^, where ROS and Nrf2 were not involved in lutein-induced upregulation of mRNAs encoding phase II antioxidant enzymes, including *sod1* and *sod2*^30^. The induction of phase II antioxidant enzymes by lutein in the absence of high levels of ROS has commonly been observed in the RPE-choroid of mice and PC12D cell line^30^, and was not specifically observed in the current study. Therefore, lutein may induce antioxidant enzyme transcription in a ROS-independent manner, although further studies will be required to elucidate the exact mechanism involved. The *sod* transcripts were not induced 18 h after light exposure in the lutein-treated group, in contrast to the vehicle-treated group, suggesting that ROS scavenging by lutein may have reduced the level to below the threshold required to induce antioxidant enzyme expression. However, mRNAs encoding these enzymes were subsequently gradually upregulated by lutein’s ROS-independent effects. Taken together, lutein may act as a scavenger in the acute phase and as an enzyme inducer in the later phase.

We further demonstrated that induction of MCP-1^9^,^12^,^17^ and macrophage infiltration^16^,^26^ after light exposure were suppressed by lutein. Narimatsu *et al*. identified macrophage infiltration in the RPE-choroid after light exposure using immunohistochemistry, consistent with the increase in *f4/80* mRNA levels observed in the RPE-choroid following light exposure^12^. Thus, the changes in *f4/80* mRNA levels in the RPE-choroid observed in the present study could reflect changes in the macrophage population within this tissue. Macrophage infiltration and changes in *mcp-1* mRNA levels occurred in parallel, suggesting that the suppressive effect of lutein on macrophage infiltration after light exposure may occur via suppression of MCP-1. We also confirmed that *mcp-1* mRNA expression was suppressed by lutein in ARPE19 cells. This could be because lutein reduced ROS levels, since MCP-1 can be regulated by ROS^12^. Lutein-mediated suppression of MCP-1 in the RPE-choroid could therefore also occur because of a local reduction in the ROS level by lutein.

Intraocular MCP-1 is reported to be present at high levels in AMD patients^31^. The impact of macrophages relates to their secretion of inflammatory cytokines, which induce both neovascularization, a characteristic finding in wet AMD, and tissue atrophy, which is observed in wet and dry AMD^32^. Lutein is taken up into the RPE, as shown previously in a study of donor eyes^33^. Thus, nutritional supplementation with lutein may have the potential to suppress MCP-1 levels and macrophage activation in the human eye. It is widely accepted that oxidative stress in the RPE is fundamental to AMD pathogenesis^34^, consistent with the use of antioxidant supplements in the AREDS formula^2^,^35^. *In vitro* analyses have shown that lutein suppressed cytokine expression induced by an oxidant, activated A2E^36^, and was cytoprotective in cells exposed to H_2_O_2_^37^. The current *in vivo* study has also contributed to improving the understanding of the effects of this nutrient supplement on the progression of AMD, although there were limitations to the current study. Firstly, there is a difference in the metabolism of xanthophylls such as lutein and zeaxanthin in mice and humans^38^. Mice have a higher activity of the xanthophyll carotenoid metabolic enzyme, β,β-carotene-9′,10′-dioxygenase (BCO2) in contrast to humans, and their xanthophyll levels are therefore lower. This means that although the RPE-choroid has higher levels of xanthophyll than those in other ocular tissues^38^ and its local lutein concentration is increased by lutein administration^39^, the effects of lutein might be underestimated in the current study. Further studies are required to determine whether the effects of oral lutein are greater in humans. Secondly, the lutein-rich marigold extract used in this study contained 8% zeaxanthin and we cannot exclude the possibility that this compound may have contributed to the effects observed. Thirdly, the immortalised ARPE19 cell line was used for some of our analyses and this may have characteristics that differ from the RPE *in vivo*. Commonly available strains of ARPE19 exhibited a heterogeneous mixture of elongated and polygonal cells, with different cellular polarities and cell-cell junctions^40^. The responses of the actin cytoskeleton, barrier function, and expression of occludin and the claudins vary according to the culture conditions^40^. Thus, it is different from the original reports of ARPE19 using passage-15 to -20 cells that was a well-established model for the *in vivo* RPE^40^.

Furthermore, additional studies are warranted before recommending lutein supplementation because the potential adverse events have not been investigated sufficiently. The xanthophyll cleavage enzyme, BCO2, regulates the tissue levels of xanthophylls and thus protects from damage related to their excessive accumulation, although BCO2 is inactivated in the human retina where high xanthophyll levels are required. In addition, the serum levels of xanthophyll reach a plateau in humans, which may prevent excessive delivery of lutein to the tissues. However, prolonged carotenoid supplementation can result in over-accumulation in the adipose tissue, as observed in the BCO2 knockout mice^41^; this suggests that tissue-related differences in BCO2 activity may determine the systemic adverse events caused by continuous lutein supplementation.

This study was performed using an acute model of light exposure, and the effect of lutein on a rate-limiting enzyme in the alternative complement activation pathway, factor D, was not evaluated. A clinical study of anti-factor D treatment for dry AMD is conducted^42^,^43^. Lutein supplementation has been reported to significantly reduce the circulating level of both factor D and its AMD pathogenesis-related products, C5a and C3d, in individuals with early AMD^44^. These findings indicate that it would be worth including evaluations of complement regulation in future studies of lutein supplementation.

Light exposure increases the risk for AMD and the present study found that it induced ROS production in the RPE and choroid, while lutein treatment reduced the local ROS levels. Lutein exhibited not only ROS scavenging activity but was also capable of inducing endogenous antioxidant SOD activity and expression. This promoted tight junction repair in the RPE, and reduced MCP-1 induction and the resulting macrophage infiltration, thus suppressing critical processes involved in AMD pathogenesis^9^,^12^,^17^,^18^. These findings will contribute to a greater understanding of the value of lutein usage in producing sustained reductions in oxidative stress necessary to prevent a chronic disease, AMD.

## Methods

### Animals

Seven- to eight-week-old male BALB/c mice (CLEA Japan, Tokyo, Japan) were housed in an air-conditioned room (22 °C) under a 12-h light/dark cycle (lights on from 8 AM to 8 PM), with free access to food and water. All animal experiments were conducted in accordance with the ARVO Statement for the Use of Animals in Ophthalmic and Vision Research, and the guidelines for the Animal Care Committee of Keio University. The experimental protocols were approved by the Animal Care Committee of Keio University (Approval Number, 08002).

### Light exposure

The light exposure experiments were performed as described previously^9^,^10^,^11^,^12^,^13^,^23^,^28^. Briefly, the mice were rested for several days and then divided into 3 groups; 2 were exposed to light and treated with either vehicle or lutein, while the control group was not exposed to light and received vehicle treatment. Prior to light exposure, mice were dark-adapted by keeping them in complete darkness for 12 h. The pupils of the mice were dilated with a mixed topical solution of 0.5% tropicamide and 0.5% phenylephrine (Mydrin-P; Santen Pharmaceutical, Osaka, Japan) just before light exposure. They were then exposed to light from a white fluorescent lamp (FHD100ECW; Panasonic, Osaka, Japan) at 2000 lux (actual measurement) for 3 h, starting at 9 AM, in a dedicated exposure box with stainless steel mirrors on each wall and on the floor (Tinker N, Kyoto, Japan). The temperature of the box was maintained at 22 °C by an air conditioner and fans; this was monitored using a thermometer. After light exposure, the mice were returned to their cages and maintained under dim cyclic light (5 lux, 12 h on/off) until they were euthanized at the indicated sampling time-points. Control mice were kept under dim cyclic light throughout and euthanized at the time of sampling.

### Lutein treatment

Each animal was administered an intraperitoneal injection of 100 mg/kg body weight lutein-rich marigold extract provided by Wakasa Seiatsu Co. Ltd. (Kyoto, Japan) or vehicle, 12 h after the light exposure. The extract was composed of 92% free and non-esterified lutein and 8% zeaxanthin. Mice that were not exposed to light were also treated with vehicle at the corresponding time point. The dose of intraperitoneally injected lutein was determined by referring to a previous report showing suppression of retinal inflammation^45^. Lutein was first dissolved in dimethyl sulfoxide (DMSO) and then diluted with phosphate-buffered saline (PBS); the injection solution contained 8% DMSO, corresponding to 24 μl per animal. Vehicle-treated animals, with or without light exposure, received the same dose of DMSO (8% DMSO in PBS). Although DMSO could be a free radical scavenger, its use was necessary because lutein is hydrophobic. It was therefore used at the lowest level possible. No mice became ill or died during these experiments. We also confirmed that this dose of DMSO did not change the data of ROS and mRNAs after light exposure by comparing the data with those from light-exposed PBS-treated mice (data not shown).

### ROS measurement

ROS were measured in the RPE-choroid complex samples as described previously^9^,^12^. Briefly, the eyes were enucleated and the cornea, lens, vitreous and retina were carefully removed; the RPE, together with the choroid, was scraped from the eyecups and placed into 100 μl PBS. The RPE and choroid could not be separated for technical reasons; therefore, RPE-choroid complex samples were used. The samples from both eyes of an individual mouse were mixed and analysed as one sample. These samples were incubated with 1 μl of 0.2 mg/ml cell-permeant fluorescent ROS detection reagent, DCFH-DA (Invitrogen, Carlsbad, CA) at 37 °C, and the fluorescence intensity was measured according to the manufacturer’s protocol, using an absorption spectrometer (Wallac ARVO SX 1420 Multilabel Counter; PerkinElmer, Waltham, MA).

### Immunohistochemistry

The eyes were enucleated, and the cornea, lens, vitreous and retina were carefully removed prior to preparing the flat-mount eyecups. The eyecups were prefixed with 4% paraformaldehyde (PFA) for 30 min and flattened by making 4 radial cuts; they were then returned to 4% PFA. The samples were blocked with TNB blocking buffer (0.1 M Tris-HCl [pH 7.5] and 0.15 M NaCl) for 30 min at room temperature, and then incubated overnight with a fluorescein isothiocyanate-conjugated anti-ZO-1 antibody (1:200; Invitrogen) and 10 μg/mL Hoechst bisbenzimide 33258 nuclear stain (Sigma-Aldrich) at 4 °C. After washing with PBS, all the samples were mounted using VECTASHIELD mounting medium H-1000 (Vector Laboratories, Burlingame, CA). Fluorescent images of the flat mounts were obtained using a confocal fluorescence microscope (FV 1000; Olympus, Tokyo, Japan). The number of intact RPE cells visible under ZO-1 immunostaining of the intracellular face of an entire cell membrane and the total RPE cells were counted in four quadrants of a 640-μm square in the central part of the retina (superior, inferior, nasal, temporal). These evaluations were conducted by observers who were blinded to the treatments.

### SOD activity

The RPE-choroid complex samples were placed in 50 μl sucrose buffer (0.25 M sucrose, 10 mM Tris, 1 mM EDTA; pH 7.4) and homogenised using a Teflon homogeniser. The samples were then centrifuged at 10,000 *g* for 60 min at 4 °C, and the supernatant was transferred to a new tube. The activity of SOD in the supernatant was determined using a SOD assay kit (Dojindo Inc., Washington, DC), according to the manufacturer’s protocol and a previous report^11^.

### Real-time reverse transcription-polymerase chain reaction (RT-PCR)

Total RNA was isolated from the RPE-choroid complex samples and the ARPE19 cell line (see below) using TRIzol reagent (Life Technologies, Waltham, MA USA). Complementary DNA was synthesised using Super-Script VILO™ Master Mix (Life Technologies) according to the manufacturer’s instructions. PCR was performed using the StepOne-Plus Real Time PCR system (Life Technologies). mRNA levels were evaluated using the ΔΔCT method and normalised to the level of glyceraldehyde 3-phosphate dehydrogenase (*gapdh*) mRNA. The mouse forward and reverse primer sequences used were *sod1,* 5′-AGACCTGGGCAATGTGGCTG-3′ and 5′-TTTATTGGGCAATCCCAATC-3′; *sod2,* 5′-CTGGACAAACCTGAGCCCTA-3′ and 5′-GAACCTTGGACTCCCACAGA-3′; *f4/80,* 5′-GAGATTGTGGAAGCATCCGAGAC-3′ and 5′-GATGACTGTACCCACATGGCTGA-3′; and *gapdh,* 5′-AACTTCGGCCCCATCTTCA-3′ and 5′-GATGACCCTTTTGGCTCCAC-3′. Mouse *mcp-1* mRNA was measured using a TaqMan gene expression assay system (*mcp-1*; Mm00441242_m1), in relation to *gapdh* expression (catalogue number 4352339E, Life Technologies). The human forward and reverse primer sequences used were *mcp-1,* 5′-AGCAAGTGTCCCAAAGAAGC-3′ and 5′-AGTCTTCGGAGTTTGGGTTTG-3′; and *gapdh*, 5′-CACCCACTCCTCCACCTTT-3′ and 5′-TCCACCACCCTGTTGCTGTAG-3′. Each experiment was performed in triplicate. Mouse primers were used for the *in vivo* experiments, and human primers were used for the *in vitro* experiments in the human ARPE19 cell line.

### Cell culture

The human ARPE19 cell line was maintained in Dulbecco’s modified Eagle’s medium (DMEM) and Ham’s nutrient mixture F-12 medium (Gibco, Carlsbad, CA) supplemented with 10% foetal bovine serum (FBS), penicillin (100 units/ml) and streptomycin (100 μg/ml) at 37 °C under a humidified atmosphere with 5% CO_2_. Lutein-rich marigold extract containing 92% lutein (Wakasa Seikatsu, Co. Ltd.) was emulsified as described previously^30^. Briefly, lutein was first diluted in a solution of ethanol (Wako Pure Chemical, Osaka, Japan) and Tween 80 (Sigma-Aldrich) (310 μl:10 μl)^30^. The ethanol was evaporated off and a micelle emulsion was formed using nitrogen gas; this was diluted in DMEM prior to addition to the culture medium^30^. The culture medium was removed and serum-free medium was added before ARPE19 cells were treated with 25 μM lutein, 50 μM lutein, or vehicle for 3 h. The cells were then washed with 1 ml PBS, sonicated in an ice bath and centrifuged at 10,000 *g* for 5 min at 4 °C prior to measuring the supernatant SOD activity, or placed in TRIzol reagent (Life Technologies) for mRNA extraction and real time RT-PCR analyses (see above).

### Statistical analysis

All results are expressed as the mean ± standard deviation. One-way analysis of variance with Tukey’s post hoc test was used to assess the statistical significance of differences, with p < 0.05 regarded as significant.

## Additional Information

**How to cite this article**: Kamoshita, M. *et al*. Lutein acts via multiple antioxidant pathways in the photo-stressed retina. *Sci. Rep.* **6**, 30226; doi: 10.1038/srep30226 (2016).
